# Depression Among Medical Students of Karachi A Cross Sectional Study

**DOI:** 10.15694/mep.2019.000181.1

**Published:** 2019-09-18

**Authors:** Ujalla Kumari, Nakeeta Dawani, Joti Devnani, Muhammad Fazal Hussain Qureshi, Fahad Khalid Soleja, Danish Mohammad, Zain Jawed Abubaker, Ayesha Haroon, Sara Sadiq

**Affiliations:** 1 Ziauddin University; 2 CMH Institute of Medical Sciences

**Keywords:** Depression, Depression score, Medical students, coping mechanism.

## Abstract

This article was migrated. The article was marked as recommended.

Introduction: Depression is a mood disorder characterized by loss of interest in daily activity, feeling of hopelessness and helplessness, decreased appetite, anger and irritability. The risk factors which leads to depression include academic demands, daily habits, sleeping hours, sedentary lifestyle, inability to cope, helplessness, increased psychological pressure, mental tension and increased work load etc. The objective of current study is to find out the prevalence of depression among students of different medical colleges of Karachi and its association with life style, habits and coping mechanisms.

Methods: A descriptive cross-sectional study was conducted in 6 months i.e. April 2018 to September 2018, using a self-designed, self-explanatory questionnaire which also included Public Health Questionnaire (PHQ-9) for identification of depression. The coefficient of reliability including Cronbach alpha was 0.839 for the questionnaire. The data was analyzed by using mean with standard deviation and frequency with percentages while association was calculated by using Chi-square test.

Results: The mean age of participants was 21.43 ±1.803. About 2/3 of the participants were female with marital status of 3/4th of participants being single. 92% of the medical students were found to be depressed while 26% of them have suicidal thoughts. Symptoms of depression were compared with depression score, which showed strong positive correlation. Depression scores were also compared with lifestyle habits of participants, including sleeping hours, exercise, recent trauma and multiple coping mechanisms, presented significant association with depression scores (p-value ≤ 0.01).

Conclusions: It is concluded that depression is highly prevalent amongst medical student populations while the lifestyle habits, sleeping hours, physical activities, recent trauma and coping mechanism showed significant positive association with depression.

## Introduction

Depression is a mood disorder characterized by loss of interest in daily activity, feeling of hopelessness and helplessness, decreased appetite, anger and irritability ( Marcus, 2012). Depressive symptoms are extremely prevalent in students nowadays which are typically characterized by sadness, loss of interest, hopelessness, nervousness or anxious feelings and even suicidal thoughts ( Kessler *et al.*, 2003). According to the results of different meta-analyses, the prevalence of depressive symptoms are higher among college students compared to the general population or non-college students ( Mikolajczyk *et al.*, 2008).

Depression is a major correspondent to the global burden of diseases and affects people all over the world with the global prevalence of 3.2% ( Moussavi *et al.*, 2007). The prevalence of depressive symptoms among college students were estimated to be 33% ( Sarokhani *et al.*, 2013) and 27-50% of medical students were found to be depressed ( Sherina, Rampal and Kaneson, 2004; Koochaki *et al.*, 2011; Rotenstein *et al.*, 2016). Looking over Pakistan, prevalence of depression and anxiety is 34% ( Mirza and Jenkins, 2004). A high prevalence of anxiety and depression 43.89% was found amongst medical students of Pakistan ( Jadoon *et al.*, 2010). The prevalence rate of depression across Karachi is 60-70% respectively ( Inam, Saqib and Alam, 2003; Khan *et al.*, 2006a). It was found that approximately 70% of the medical students suffered from anxiety and depression in Karachi ( Khan *et al.*, 2006b).

A number of studies have reported a high prevalence of depression and anxiety among medical students, both internationally and in Pakistan. And risk factors which lead to depression; such as academic demands, exams, inability to cope, helplessness, increased psychological pressure, mental tension and high work load etc. ( Shaikh *et al.*, 2004; Khan *et al.*, 2006a). It is estimated that by the year 2020, anxiety and depression will be the second most common cause of disability worldwide ( Lopez and Murray, 1998). Stress during education can lead to mental distress and have a negative impact on cognitive functioning and learning. Hence, there is a need to quantify the anxiety, depression and its associated factors among medical students for their counselling and rehabilitation ( Khan *et al.*, 2006a; Qureshi *et al.*, 2019).

The objective of current study is to find out the prevalence of depression among students of different medical colleges of Karachi and its association with symptoms, life style habits and coping mechanisms.

## Methods

A descriptive cross-sectional study was conducted using a self-designed, self-explanatory questionnaire Public Health Questionnaire (PHQ-9) of Pfizer ( Zhang *et al.*, 2013) for identification of depression. Questionnaire includes questions related to demographics i.e. age, gender, ethnicity, year of study, lifestyle, coping mechanisms for depression. The questionnaire was validated by pilot testing on a sample of 20 students. The coefficient of reliability including Cronbach alpha was 0.839 for the questionnaire. The questionnaire was disseminated personally; names of students were not recorded. Ethical approval certificate was obtained by Ziauddin University which was submitted and accepted by all the institutions participating in research. Sample Size was calculated using Openepi and was found to be 200.

Study was conducted in 6 months i.e. April 2018 to September 2018. Multistage sampling technique was done, in first stage three private medical colleges (Ziauddin University, Bahira University, Jinnah Medical and Dental College) and three government medical Colleges (DOW University, Jinnah Sindh Medical University, Karachi Medical and Dental College) were selected, while in the second stage students from those universities/colleges were randomly selected for the study. Students from all years like first, second, third, fourth and fifth year of MBBS were included. Students of BDS, Pharmacy, and Physiotherapy etc. were excluded to avoid bias.

The data was analyzed by using Statistical Package for Social Sciences (SPSS-22). The mean with standard deviation were calculated for quantitative variables while frequency and percentages for qualitative variables. The association of depression with appeared symptoms, life style habits and coping mechanisms were calculated by using Chi-square test.

## Results/Analysis

About 230 questionnaires were distributed in different public and private sector medical universities of Karachi among MBBS students in different years of study, out of which 200 were fully filled. Data was collected from 3 government and 3 private sector universities of Karachi. Depression was identified using Public Health Questionnaire 9 (PHQ-9). Majority of the participants were suffering from mild depression, 92% of the population was suffering from some degree. 41% of population was suffering from Mild depression, 27% from minimal depression, 14% from moderate depression, 7% from moderately severe depression and 3% from severe depression. 8% population was not suffering from depression at all. While 26% of them also had suicidal intentions. The mean age of participants was 21.43 with standard deviation of 1.803. About 2/3 participants were female with marital status of 3/4 ^th^ of participants being single. Considering ethnicity majority of study participants were Panjabi speaking. Students from each year were equally included in the study. The basic demographic characteristics of participant are shown in Table 1.

**Table 1.** Characteristics of study Participants

	n	%
Gender		
Male	67	35.5
Female	133	65.5
Marital Status		
Single	170	85
Committed	22	11
Married	05	2.5
Divorced	03	1.5
Ethnicity		
Sindhi	29	14.5
Punjabi	54	25
Pathan	22	11
Baloch	04	2
Urdu Speaking	73	36.5
Others	18	9
Year of Study		
First Year	40	20
Second Year	40	20
Third Year	40	20
Fourth Year	40	20
Fifth Year	40	20

Symptoms of depression like hopelessness, helplessness, loss of interest in daily activities, appetite or weight changes, sleep changes, anger or irritability, loss of energy, self-loathing, reckless behaviour, concentration problems and unexplained aches and pains were compared with depression score, including minimal, mild, moderate, moderately severe and severe depression, which showed strong positive correlation as p-values were highly significant (p-value ≤ 0.01), mentioned in Table 2.

**Table 2.** Compression of Depression Scores with Depression Symptoms

Symptoms	Minimal Depression		Mild Depression		Moderate Depression		Moderately Severe Depression		Severe Depression		No Depression		P Value
	n	%	n	%	n	%	N	%	n	%	N	%	
Hopelessness	11	5.5	36	18	13	6.5	10	5	4	2	1	0.5	0.000
Helplessness	19	9.5	39	19.5	16	8	12	6	6	3	3	1.5	0.000
Loss of interest in daily activities	12	6	50	25	16	8	13	6.5	4	2	3	1.5	0.000
Appetite or weight changes	17	8.5	32	16	14	7	9	4.5	4	2	3	1.5	0.038
Sleep changes, Anger or irritability, Loss of energy, Self-loathing, Reckless behaviour, Concentration problems	30	15	54	27	21	10.5	14	7	5	2.5	5	2.5	0.001
Unexplained aches and pains	13	6.5	24	12	10	5	10	5	4	2	1	0.5	0.001
None of the Symptoms	8	4	5	2.5	1	0.5	0	0	0	0	4	2	0.055

Depression scores were compared with lifestyle habits of participants and some of the habits, including sleeping hours, exercise, and recent trauma showed significant relation with depression scores (p-value ≤ 0.01). Looking over the multiple coping mechanisms, significant association was noted in minimizing the symptoms as either talking to a friend or family member, sleeping or meditation. On the other hand, ignoring the issues or optimism didn’t show any significant association as shown in Table 3.

**Table 3.** Compression of Depression Scores with Lifestyle

Variable	Minimal Depression		Mild Depression		Moderate Depression		Moderately Severe Depression		Severe Depression		No Depression		P Value
	n	%	n	%	n	%	n	%	n	%	N	%	
Sleeping Hours													
2-4 Hours	0	0	7	3.5	2	1	3	1.5	0	0	1	0.5	0.000
4-6 Hours	25	12.5	37	18.5	18	9	6	3	0	0	5	2.5	
6-8 Hours	27	13.5	32	16	4	2	1	0.5	3	1.5	9	4.5	
>8 Hours	3	1.5	6	3	3	1.5	4	2	3	1.5	1	0.5	
Exercise													
Daily	15	7.5	8	4	3	1.5	1	0.5	1	0.5	3	1.5	0.328
Alternative Days	9	4.5	21	10.5	5	2.5	2	1	1	0.5	5	2.5	
Weekly	15	7.5	24	12	9	4.5	3	1.5	1	0.5	6	3	
Never	16	8	29	14.5	10	5	8	4	3	1.5	2	1	
Free Time													
Sleeping	9	4.5	28	14	11	5.5	8	4	2	1	3	1.5	0.025
With Friends and Family	36	18	32	16	15	7.5	2	1	1	0.5	12	6	0.000
Alone	7	3.5	18	9	7	3.5	5	2.5	3	1.5	1	0.5	0.081
Watching TV or Using computer	23	11.5	41	20.5	12	6	7	3.5	4	2	4	2	0.429
Sports	6	3	9	4.5	3	1.5	1	0.5	0	0	3	1.5	0.858
Reading	11	5.5	10	5	2	1	1	0.5	0	0	3	1.5	0.445
Coping Mechanisms													
Talking to a friend or family	30	15	34	17	13	6.5	3	1.5	0	0	8	4	0.047
Sleeping	14	7	39	19.5	14	7	10	5	2	1	2	1	0.002
Ignoring	11	5.5	20	10	1	0.5	1	0.5	0	0	3	1.5	0.543
Optimism	10	5	19	9.5	8	4	3	1.5	3	1.5	3	1.5	0.126
Meditation	6	3	6	3	2	1	0	0	1	0.5	5	2.5	0.026
Recent Trauma													
Death Of a family member	13	6.5	19	9.5	10	5	5	2.5	1	0.5	4	2	0.673
Death of a friend or pet	5	2.5	9	4.5	6	3	3	1.5	1	0.5	0	0	0.246
Harassment	2	1	7	3.5	5	2.5	5	2.5	3	1.5	0	0	0.000
Social Embarrassment	5	2.5	14	7	8	4	5	2.5	2	1	1	0.5	0.049
Failure to solve problem	11	5.5	25	12.5	9	4.5	10	5	4	2	2	1	0.001

## Discussion

Depression is a significant problem in medical schools. The overall depression rate in medical students of Karachi is found to be 92%, whereas it was previously recorded at 70% in a study ( Khan *et al.*, 2006a). The result shows 41% of students are mildly depressed and 10 percent severely or close to severely depressed. Similarly, in a meta-analysis for depression, about 27% of medical students suffered from depression ( Rotenstein *et al.*, 2016). In addition, 11.1% of students suffered from suicidal ideation. This statistic is very close to the 10% of individuals in the data presented. A study on medical students in Egypt also pointed out that 65% of students were depressed ( Fawzy and Hamed, 2017). This shows that there is a repeated trend of depression in many medical schools. An article from Cameroon shows that 30.6% of their medical students had major depressive disorder ( Ngasa *et al.*, 2017). This again shows a repeated trend of depression. An article by Lane *et al*.,2016 states that Pakistani American ethnicity has a non-significant trend in depression ( Lane, Cheref and Miranda, 2016). However, the results stated earlier do not correlate with the population in Pakistan medical schools. Therefore, depression is an issue.

Our study suggests that certain lifestyle practices, coping mechanisms, and life events can have an effect on depression. Specifically, sleep is a highly significant factor towards depression. Likewise, in one study on undergraduates in Nepal, 35.4% of students were sleep deprived ( Bhandari *et al.*, 2017). Out of that percentage of students 21.2% of students were depressed. Sleep deprivation is a common correlate towards depression. Another study amongst adolescents stated that sleep deprivation increased the risks of depression between 25-38% ( Roberts and Duong, 2014). Therefore, this minor finding may signify that students are depressed due to sleep deprivation.

Another finding statistically significant was spending time with friends and family. It seems that social isolation is a keen risk factor towards depression. However, our statics for being alone did not specify any significance. This may be because according to Matthews et al, socially isolated young adults are not necessarily lonely. It is the feeling of loneliness which is a risk factor for depression ( Matthews *et al.*, 2016). While coping with family and friends can reduce the risk of depression.

Another variable that has a great significance is, any recent trauma. Specifically, the study suggests harassment, social embarrassment, and failure to solve problems as the main links. It seems that in the case of harassment, workplace bullying and sexual harassment are further correlates of stress ( Taniguchi *et al.*, 2016) and the current study shows significant association with some of the factors of recent trauma.

Depression is highly prevalent amongst medical student populations. Therefore, actions must be taken to figure out the causes and solutions to these problems. If harassment is the cause of depression then in the future we must further investigate what kind of harassment is going on, and then the actions to prevent it. We cannot pinpoint what failures to problems causes’ depression. One article suggests that board examination failure can play a role ( Bhandari *et al.*, 2017). Moreover Silva *et al.*, 2017 state that higher academic correlate with improvement of depression ( Silva *et al.*, 2017). If that is the cause then certain programs need to come up with facilities to help students cope with these initial stressors as is mentioned by one of the study which highlighted the medical student syndrome (MSS) as one of the major stressor ( Sadiq, Majeed and Khawar, 2018).

Current study specifically tried to take out the prevalence of depression amongst medical students. The study did its best to figure out daily symptoms and coping mechanisms, however, these specific coping mechanisms could not be further investigated. Future recommendation is to conduct a larger scale study across Pakistan to highlight the prevalence and cause of depression among medical students.

## Conclusion

It is concluded that depression is highly prevalent amongst medical student populations while the lifestyle habits, total sleeping hours, physical activities, recent trauma and coping mechanism showed significant positive association with depression. As the medical students are highly vulnerable to depression, actions must be taken to figure out the causes and solutions to their problems.

## Take Home Messages

• It is concluded that depression is highly prevalent amongst medical student.
• Lifestyle habits, total sleeping hours, physical activities, recent trauma and coping mechanism showed significant positive association with depression.
• As the medical students are highly vulnerable to depression, actions must be taken to figure out the causes and solutions to their problems.

## Notes On Contributors

Miss Ujalla Kumari: Conceptualized and designed this study, both the intervention described and the rigorous measurement and analysis; spearheaded the acquisition of data and helped in the analysis and interpretation of the collected data; revised the drafted article critically for important intellectual content; provided final approval of the version to be published. She is currently a third year Medical Student doing MBBS from Ziauddin University.

Miss Nakeeta Dawani: Conceptualized and designed this study, both the intervention described and the rigorous measurement and analysis; spearheaded the acquisition of data and helped in the analysis and interpretation of the collected data; revised the drafted article critically for important intellectual content; provided final approval of the version to be published. She is currently a third year Medical Student doing MBBS from Ziauddin University.

Miss Joti Devnani: Conceptualized and designed this study, both the intervention described and the rigorous measurement and analysis; spearheaded the acquisition of data and helped in the analysis and interpretation of the collected data; revised the drafted article critically for important intellectual content; provided final approval of the version to be published. She is currently a third year Medical Student doing MBBS from Ziauddin University.

Mr Muhammad Fazal Hussain Qureshi: Conceptualized and designed this study, both the intervention described and the rigorous measurement and analysis; spearheaded the acquisition of data and helped in the analysis and interpretation of the collected data; revised the drafted article critically for important intellectual content; provided final approval of the version to be published. I am currently a third year Medical Student doing MBBS from Ziauddin University.

Mr Fahad Khalid Soleja: Conceptualized and designed this study, both the intervention described and the rigorous measurement and analysis; spearheaded the acquisition of data and helped in the analysis and interpretation of the collected data; revised the drafted article critically for important intellectual content; provided final approval of the version to be published. He is currently a third year Medical Student doing MBBS from Ziauddin University.

Mr Danish Mohammad: Conceptualized and designed this study, both the intervention described and the rigorous measurement and analysis; spearheaded the acquisition of data and helped in the analysis and interpretation of the collected data; revised the drafted article critically for important intellectual content; provided final approval of the version to be published. He is currently a third year Medical Student doing MBBS from Ziauddin University.

Mr Zain Jawed Abubaker: Conceptualized and designed this study, both the intervention described and the rigorous measurement and analysis; spearheaded the acquisition of data and helped in the analysis and interpretation of the collected data; revised the drafted article critically for important intellectual content; provided final approval of the version to be published. He is currently a third year Medical Student doing MBBS from Ziauddin University.

Miss Ayesha Haroon: Conceptualized and designed this study, both the intervention described and the rigorous measurement and analysis; spearheaded the acquisition of data and helped in the analysis and interpretation of the collected data; revised the drafted article critically for important intellectual content; provided final approval of the version to be published. She is currently a third year Medical Student doing MBBS from Ziauddin University.

Dr Sara Sadiq: Conceptualized and designed this study, both the intervention described and the rigorous measurement and analysis; spearheaded the acquisition of data and helped in the analysis and interpretation of the collected data; revised the drafted article critically for important intellectual content; provided final approval of the version to be published. She is Assistant Professor working in Department of Physiology.
