# Supplementary material for: Depression Among Medical Students of Karachi A Cross Sectional Study
Source: MedEdPublish (2016). 2019 Sep 18;8:181. [Version 1] doi: 10.15694/mep.2019.000181.1 (PMC10712523; doi:10.15694/mep.2019.000181.1)
Supplement: Peer review history

# Reviewer report: Reviewer response for version 1

**DOI:** 10.21956/mep.19904.r30140

**Published:** 2019-10-05

**Authors:** Lubna Jahanzeb

**Affiliations:** 1 Baqqai Medical University

This review has been migrated. The reviewer awarded 5 stars out of 5 It is very overwhelming to see this student based research on such an important topic. Being a teacher in Pakistani educational setup for more than ten years help me understand this paper in detail. Introduction is very well written with adequate references to support study. Methodology is explained in a very simple yet interesting manner and can help other researcher’s to replicate this study easily. The most interesting part of paper is results and discussion which make the argument more interesting and gives the reader perfect picture of the current scenario and reasons behind this issue.I highly recommend this paper.

Reviewer Expertise:

NA

No decision status is available

# Reviewer report: Reviewer response for version 1

**DOI:** 10.21956/mep.19904.r30136

**Published:** 2019-10-04

**Authors:** Stijntje Dijk

**Affiliations:** 1 Erasmus Medical Center

This review has been migrated. The reviewer awarded 2 stars out of 5 The topic of this paper, mental wellbeing among medical students, is very relevant. The study seeks to identify the prevalence of depression among students in a local setting, which provides valuable information to a field in which cited prevalence vary strongly across literature, and could provide an incentive and starting point for interventions in Karachi. The fact that the paper is written mainly by medical students themselves is commendable.We, however, do have several reservations regarding this paper that we would encourage the authors to consider if submitting a revised paper.- Comparability: Seeing as the main focus of the paper is investigating and comparing the prevalence of depression, it is crucial that the cited literature in both the discussion and introduction is comparable. A number of prevalence papers are cited, however, without the context of which instruments and cutoff values were used, and who these groups were, the reader may be led to draw incorrect conclusions. For example, the authors compare the 92% prevalence of depression among medical students in Karachi to the previously recorded 70% in the study by Khan et al, however do not describe that Khan et al. used a different instrument (AKUADS) with a much higher cutoff value (19) compared to PHQ-9 with a cutoff value of 1, which states that students have a minimal depression even if they feel tired several days a week alone. One should also note the limitation that the PHQ-9 is a self-report tool that should be verified by a clinician prior to a definitive diagnosis being made. Several other citations on current prevalence rates are from 15 years ago, whereas we’d be curious to hear about more recent developments. The paper could benefit from providing more detailed information on the comparability across studies.- Reproducibility of methods: The authors stated they used a self-designed, self-explanatory questionnaire that contains the PHQ-9, however, have not explained in what ways they have modified the existing and validated instrument. The authors used the tool Openepi to conduct statistical calculations however have not provided the reader with a level of detail that would allow us to replicate their results and calculations, which we encourage the authors to add in any revisions.- Results: We found it difficult to interpret tables 2 and 3 and wondered whether there might be alternative formats that could demonstrate the point the authors wish to make. We additionally caution that the discussion then makes causal assumptions based on correlation, whereas for example, the correlation found between lack of sleep leading to depression may also have been depression leading to lack of sleep, as is also the case for the conclusions drawn on isolation and physical activity. The results on suicidal ideation are expressed differently in the abstract (26% of students with depression) and the conclusion (10% of all participants). We believe that the paper would benefit from aligning abstract and body, and should provide the reader with an explanation on what cutoff value is used for suicidal ideation.We thank the authors for their valuable work and interesting paper and hope they would be able to address some of our reservations in any revised versions.

Reviewer Expertise:

NA

No decision status is available

# Reviewer report: Reviewer response for version 1

**DOI:** 10.21956/mep.19904.r30137

**Published:** 2019-09-27

**Authors:** BALAJI ARUMUGAM

**Affiliations:** 1 TAGORE MEDICAL COLLEGE AND HOSPITAL

This review has been migrated. The reviewer awarded 4 stars out of 5 This study is the need of the hour among medical students. As a medical teacher for the past 15 years, I have come across many students having suicidal tenancies and I have sent them for psychiatric counselling. And this year world mental health day them is on suicide prevention, to focus more on 40 seconds action. After completing a debate and a panel discussion in CME programme yesterday in our institution, reading this article and getting to know that the 26% had suicidal thoughts is really worrying and alarming. This descriptive study was conducted nicely and the results are projected clearly. Please kindly consider the depressed students to have some continuous counseling sessions and the treatment and follow up.

Reviewer Expertise:

NA

No decision status is available

# Reviewer report: Reviewer response for version 1

**DOI:** 10.21956/mep.19904.r30139

**Published:** 2019-09-26

**Authors:** Rehana Faryal Mehdi

**Affiliations:** 1 Ziauddin University

This review has been migrated. The reviewer awarded 5 stars out of 5 Indeed a very insightful analysis of one of the major condition encountered by students, reading this paper gives me a complete picture of the situation and measures taken. It is interesting to see that this study was conducted and led by students.I will recommend this study to every researcher working in this area.

Reviewer Expertise:

NA

No decision status is available

# Reviewer report: Reviewer response for version 1

**DOI:** 10.21956/mep.19904.r30138

**Published:** 2019-09-25

**Authors:** Trevor Gibbs

**Affiliations:** 1 AMEE

This review has been migrated. The reviewer awarded 3 stars out of 5 Wellness and illness in medical students is a very important topic, which many medical schools, despite their best efforts, are not really coping with. This makes this paper an interesting one to read, covering such an important area. I was pleased to see that it was the students who had developed and written this paper.I am not sure that many of the papers findings are new to the readers with interest in this area, but basing their research in Pakistan does give it a different perspective. The authors have researched their paper well as seen by the large number of relevant references, and I believe have taken a logical and appropriate approach to their research. I personally would have liked to see more description of activities than the papers concentration on statistics and I was left wanting in knowing more about the second half of the study looking at coping mechanisms, particularly given that most of the authors were students. With the results showing as they are, then clearly one must look at how we manage this problem, specifically as it seems that approximately a quarter of the students had suicidal ideas. I do not think that the authors need to make this a national study, I hope that they now have enough material to extend this study into the areas of prevention and management.I would recommend this paper to those involved in student support and curriculum planning

Reviewer Expertise:

NA

No decision status is available
